# A qualitative and quantitative study of self-reported positive characteristics of individuals with ADHD

**DOI:** 10.3389/fpsyt.2022.922788

**Published:** 2022-10-12

**Authors:** Lessa M. Schippers, Lisa I. Horstman, Hans van de Velde, Rob Rodrigues Pereira, Janneke Zinkstok, Jeanette C. Mostert, Corina U. Greven, Martine Hoogman

**Affiliations:** ^1^Department of Human Genetics, Radboud University Medical Center, Nijmegen, Netherlands; ^2^Donders Institute for Brain, Cognition and Behavior, Radboud University, Nijmegen, Netherlands; ^3^ADHD-Europe, Chair Science Committee Adult Patient Organization ADHD, Dyslexia and Dyscalculia, Impuls & Woortblind, Nijkerk, Netherlands; ^4^Medical Center Kinderplein, Rotterdam, Netherlands; ^5^Dutch Adult Patient Organization ADHD, Dyslexia and Dyscalculia, Impuls & Woortblind, Nijkerk, Netherlands; ^6^Department of Psychiatry, Radboud University Medical Center, Nijmegen, Netherlands; ^7^Karakter Child and Adolescent Psychiatry University Center, Nijmegen, Netherlands; ^8^Department of Psychiatry, University Medical Center Utrecht, Utrecht, Netherlands; ^9^Department of Cognitive Neuroscience, Radboud University Medical Center, Nijmegen, Gelderland, Netherlands; ^10^King's College London, Institute of Psychiatry, Psychology and Neuroscience, Social, Genetic and Developmental Psychiatry Centre, London, United Kingdom

**Keywords:** ADHD, strengths, neurodiversity, qualitative research, neurodevelopmental disorders, participatory research

## Abstract

Research in Attention-deficit/hyperactivity disorder (ADHD) has had a clear focus on treatment and the dysfunction in specific situation associated with the condition. However, self-report, observational and anecdotal evidence indicates that there are also positive aspects associated with ADHD. Research on the potential positive features in individuals with an ADHD diagnosis is still limited, especially studies with larger representative samples. Here we performed qualitative research to identify positive aspects and strengths associated with ADHD in a large convenience sample from the Dutch organization for people with ADHD, dyslexia and dyscalculia. We sent out open-ended questionnaires to the members of the organization, asking what they consider to be positive aspects of their ADHD. From the responses of individuals with ADHD (*n* = 206), we extracted 116 codes, which were assigned to thirteen subthemes, which in turn led to five themes. These themes were: *Creativity, Being dynamic, Flexibility, Socio-affective skills*, and *Higher-order cognitive skills*. Core symptoms of ADHD such as impulsivity and hyperactivity were also considered positive aspects of ADHD by a minority of participants. After showing our results to a group of additional individuals with ADHD (focus group) they confirmed the identified positive aspects of ADHD. They also helped us with the interpretation of our findings and mentioned certain positive aspects to be a consequence of living with ADHD (being open minded and being honest). In conclusion, experiencing positive aspects seems to be common in ADHD as almost all participants reported positive aspect of ADHD, these aspects cover many different domains. Awareness about ADHD's strengths might help individuals with ADHD and their environment to better cope with, accept or embrace their diagnosis and for example make educational or occupational choices that fit them well. To incorporate these positive aspects in the understanding of ADHD, future research should focus on quantifying strengths in ADHD, and on investigating the link between these aspects and clinical characteristics and how this new knowledge can be implemented in psychoeducation and find its way to education and occupational settings.

## Introduction

Attention-deficit/hyperactivity disorder (ADHD) is a common neurodevelopmental condition, occurring in 5.9% of children (1). ADHD symptoms and ADHD as a diagnosis may persist across the lifespan, and ADHD diagnosis is present in about 2.5% of adults (2, 3). ADHD is characterized by age-inappropriate levels of hyperactivity, impulsivity and inattention (4). Individuals with ADHD may present with cognitive deficits, such as altered reward processing, deficient time estimation, and executive dysfunction (5, 6). These cognitive deficits are associated with problems in daily life, for example problems with executive functioning have been related to functional impairment at work (7, 8). In addition to these behavioral symptoms and cognitive impairments, individuals with ADHD often present with low self-esteem, academic underachievement and unemployment, all resulting in reduced quality of life (9–13).

That ADHD may be associated with positive aspects, and/or strengths, is relatively unknown and an often neglected aspect in previous scientific literature. Recently, positive aspects of ADHD have gained attention. This fits well with a conceptual shift that is taking hold in the field of neurodevelopmental conditions, and is being championed by caregivers and individuals with those conditions. Slowly our society is starting to focus more on embracing diversity rather than excluding individuals with a diagnosis. “Neurodiversity” is a term that is often linked to this conceptual shift. One key assumption of neurodiversity is that all forms of neurological diversity might be valuable, depending on the environment, and that this diversity may also have some selective and evolutionary advantages (14, 15).

Although research into positive aspects of ADHD is scarce, three studies aimed to study positive aspects associated with ADHD. Mahdi and colleagues performed a qualitative study to investigate abilities and disabilities in individuals with ADHD in five different countries, by organizing focus groups and interviews with in total 76 individuals with ADHD, and other stakeholders (parents, care givers, health professionals) (16). In their study, they found that individuals with ADHD regard the following aspects of ADHD as positive: a high level of energy and drive, creativity, hyperfocus, agreeableness, empathy, and willingness to assist others (16). Sedgwick and colleagues specifically investigated positive aspects of ADHD. They performed in-depth interviews with six successful adult men with ADHD to investigate what they consider positive aspects of ADHD (17). In their study, they linked positive aspects of ADHD to six core themes: dynamism, courage, energy, humanity, resilience and transcendence (17). Holthe and Langvik interviewed five successful women with ADHD to capture the experiences of women with ADHD, both positive and negative (18). They found creativity, determination, ability to get easily interested and excited about new things, adventurousness, and willingness to take risks as positive aspects that these successful women related to their ADHD (18).

In addition to the qualitative research, there is also quantitative evidence for a link between the positive traits mentioned by individuals with ADHD and the ADHD phenotype. Most of these studies focused on creativity. Creativity is a broad concept and can be divided into different aspects such as convergent and divergent thinking and creative achievements. Divergent thinking, which entails coming up with different solutions for one problem, was found to be associated with ADHD (19). In addition to creativity, hyperfocus and sensory processing sensitivity are other traits that have been positively associated with ADHD in quantitative studies (20, 21).

Although the qualitative studies conducted so far offer a great and much needed exploration into the positive aspects of ADHD, they rely heavily on studies in successful adults with ADHD (17, 18). To more comprehensively capture the diversity and universality of positive characteristics of ADHD experienced by individuals with ADHD, we need larger and more diverse samples. This is also of importance for directing future quantitative studies of positive aspects of ADHD, which are needed to determine if the self-reported positive aspects are indeed more present in people with ADHD so that they can be included into the management of ADHD and education about ADHD. Therefore, in this current project, we aim to increase the knowledge body on potential positive characteristics of ADHD to provide a more complete picture of the ADHD phenotype. Here, we report a qualitative online survey study with open questions among members of the Dutch organization for ADHD, dyslexia, and dyscalculia, and focus groups to discuss findings and incorporate participants' views. In addition, we performed quantitative analyses on the number of positive aspects reported in relation to age, gender, employment, and treatment.

## Methods

### Participants and procedure

All members of the Dutch organization for people with ADHD, dyslexia or dyscalculia (Impuls & Woortblind) were invited *via* email to anonymously fill out an online questionnaire. The group of members (*n* = 1,326) consists of individuals with an ADHD diagnosis, and/or dyslexia, and/or dyscalculia, and partners and parents of individuals with these classifications. The invitation to participate in the survey was explicitly directed toward individuals with ADHD/dyslexia/dyscalculia and not their relatives. In the survey participants were asked if they have a current ADHD diagnosis (yes/no) and a dyslexia/dyscalculia diagnosis (yes/no). They were also requested to provide basic demographic information about their age, sex, their diagnosis (ADHD/ADD yes/no, dyslexia/dyscalculia yes/no), employment status and about their treatment by a psychiatrist/psychologist or coach (yes currently/no/not anymore/other). Participants were asked to freely report what they considered positive aspects of ADHD and in a subsequent question, what they considered negative aspects of ADHD, with unlimited time. The questionnaire was available between the 15th of November 2019 and the 20th of February 2020. SurveyMonkey was used to collect the data. As our current study had the specific aim to study positive aspects of ADHD, we only used the data from the participants with an ADHD diagnosis.

### Qualitative analysis

We extracted the data from SurveyMonkey and imported the output of the open-ended question about the positive aspects of ADHD in ATLAS-ti 9.1.6. The data consisted of answers of one word, such as *creative, empathic, happy* or *enthusiastic* or quotes such as “always up for something”. The initial analysis consisted of coding those words and quotes, executed by two raters (MH and AOB). The codes are considered the smallest units in the data. In most instances the actual word corresponded with the given code, but in some cases we made a combination of descriptions that were closely linked. For example, when data mentioned “associative”, or “making connections easily”, or “making links quickly” we gave all of these the code: “associative thinking/seeing connections”. Discrepancies between the two coders were resolved by discussing them and deciding on the best code to fit the answer.

**Figure 1:**
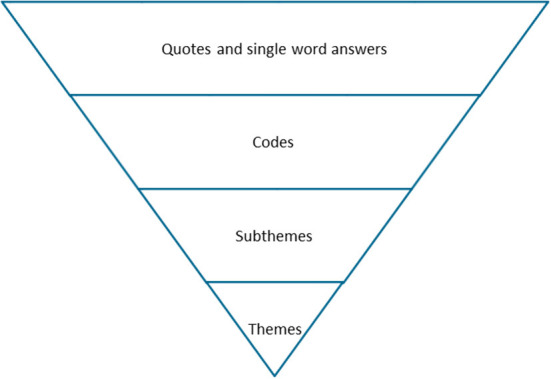
Schematic overview of the qualitative steps of our analysis.

After the coding process LS, LH, JM, CG, MH discussed the codes and how they could be grouped into subthemes. This was an iterative process and evaluations were done multiple times by discussing the subthemes. Although some codes fit in multiple subthemes, we decided to use the codes only once in the subtheme grouping. In the final step, a similar process was executed to form the themes, please see Figure 1 for a visual representation of the method.

**Figure 2:**
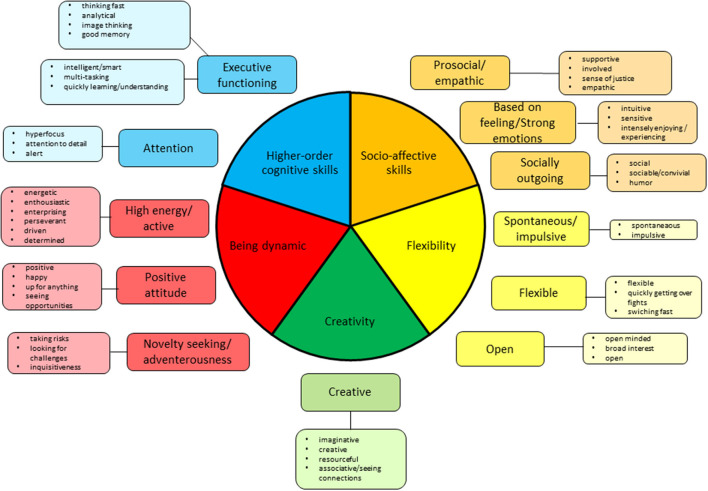
Overview of the self-reported positive aspects of ADHD grouped into subthemes and themes.

After we finalized the subthemes and themes, we validated the identified subthemes and themes from our analysis in a focus group session. To recruit participants for this focus group, we placed an advertisement on the website of the Dutch organization for people with ADHD, dyslexia and dyscalculia. These participants had not participated in the original online survey. We presented (Figure 2) to the group and asked them if our subthemes were representative of positive aspects related to ADHD. They were also asked if they thought certain positive aspects were missing. After presenting the figure we asked them how they thought these results could be used in the future.

### Quantitative analysis

In addition to the qualitative analysis, we performed exploratory quantitative analysis to further our understanding of the qualitative results. We explored the relationship between the total number of codes that were mentioned by each individual and basic characteristics such as age and sex, by performing Pearson's correlations and *t*-test, respectively. For sex, we also investigated sex differences per theme by calculating per theme how many men and how many women have reported at least one positive aspect. These are transformed to ratios (i.e., number of men reporting at least one aspect in Theme 1/total number of men). The ratios for men and women were compared by performing a Chi-square test, for each theme separately. We used a Bonferroni correction to counteract multiple comparison problems, using a significance threshold of *p* < 0.01 (0.05/5 themes = 0.01).

To investigate whether employment status was associated with the number of positive aspects mentioned, we performed a group analysis controlling for age and sex and compared individuals who were in employment or were receiving education, with individuals who were unemployed (operationalized by grouping individuals receiving unemployment or disability benefits) on the number of positive codes mentioned. Finally, to learn more about the role of treatment on positive aspects in ADHD, we compared the group of people who received treatment (current or past) with those who were not, or had not been in treatment, on the total number of codes mentioned.

## Results

### Sample characteristics

Of the 1,326 members of the Dutch organization for people with ADHD, Impuls & Woortblind, that were sent an email with the invitation to participate, 346 members completed the questionnaire, a response rate of 26%. As we aimed to learn more about the positive aspects of ADHD from individuals with ADHD themselves for this project, we included data from only those with an ADHD diagnosis and not those with dyslexia/dyscalculia without ADHD or those who were relatives of individuals with ADHD. This resulted in excluding 49 individuals who had a diagnosis of dyslexia/dyscalculia without ADHD and 45 individuals who were a family member or a partner of somebody with ADHD but with no current ADHD diagnosis themselves. Participants who did not answer the question about their experiences of positive and negative aspects of ADHD (*n* = 46) were excluded from qualitative and quantitative analyses because the reasons for leaving these fields blank were unclear. Therefore, the data of 206 participants were included in our final analysis. A full overview of the demographics of the study sample are shown in Table 1. The mean age was 46.61 (*SD* = 12.81). There were more women (*n* = 129) than men (*n* = 77) participating in the study and a minority of the study sample had co-existing dyslexia/dyscalculia. A large portion of our sample reported being employed, with unemployment plus disability benefits rates being around 13%. Five participants explicitly reported having no positive aspects related to their ADHD diagnosis (“I don't see any benefits in my case”) and eight participants reported no positive aspects. These subjects were not part of our qualitative analysis, but were part of our quantitative analysis. One participant noted that all aspects of ADHD can be considered as positive without elaborating on these positive aspects. Due to lack of information, we could not incorporate this answer in our qualitative nor in our quantitative analyses.

**Table 1:** Demographic characteristics of the study sample.

**Participants with ADHD**	**Total**	**Males**	**Females**	***p*-value for differences between males and females**
*n* (%)	206 (100)	77 (37.4)	129 (62.6)	0.67
Average age (SD) *	46.61 (12.81)	47.12 (13.95)	46.31 (12.13)	0.11
Co-occurring Dyslexia/dyscalculia *n* (%)l	32 (15.5)	8 (10.4)	24 (18.6)	0.66
(missing = 10)				
Employment *n* (%)				
Employed^†^	126 (61.2)	45 (58.4)	81 (62.8)	0.48
Unemployed^†^	8 (3.9)	4 (5.2)	4 (2.6)	0.3
Student^†^	9 (4.4)	5 (6.5)	4 (3.1)	0.11
Retired^†^	7 (3.4)	5 (6.5)	2 (1.6)	0.11
Disability pension^†^	19 (9.2)	5 (6.5)	14 (10.9)	0.16
Stay-at-home parents^†^	5 (2.4)	0 (0)	5 (3.9)	0.7
Other^†^	32 (15.5)	13 (16.9)	19 (14.7)	0.76
Treatment *n* (%)				
Current treatment	67 (32.5)	28 (36.4)	39 (30.2)	0.88
Past treatment	70 (34.0)	29 (37.7)	41 (31.8)	0.73
No treatment	47 (22.8)	17 (22.1)	30 (23.3)	1
Other	1 (0.5)	0 (0)	1 (0.08)	
(missing = 21)				

The employment “other” group consisted of combinations of the different categories; employed + student (*n* = 5), employed + disability pension (*n* = 5), employed + stay- at- home parent (*n* = 8), employed + unemployed (*n* = 3), unemployed + disability pension (*n* = 2), employed + retired (*n* = 3), retired + other (*n* = 3), and other such as; does not have to work (*n* = 1), other, not specified (*n* = 2).

*T-test.

^†^Fisher's exact test.

### Qualitative results

Coding all the answers of the participants resulted in a list of 116 codes. Among those 116 codes, the reporting frequencies had a large range, from 1 to 159. In further subgrouping those codes into subthemes and themes, we excluded low frequency codes (<5 reports), as these represent positive aspects experienced by only a few participants. A list of low frequency codes can be found in Table 2. A total of 54 codes which had 5 or more reports, were assigned to 13 subthemes and 5 core themes (Figure 2). A list of these 54 codes and their corresponding frequencies can be found in Table 3. The following themes were formed and described below: Creativity, Being dynamic, Flexibility, Socio-affective skills and Higher-order cognitive skills. Given the qualitative nature of the analysis, we present the themes in random order and will not mention frequencies.

**Table 2:** List of low frequency codes excluded from the qualitative analysis.

**Codes**	**Frequency**
Conceptual thinking	4
Taking initiative	4
Innovative	4
Colorful	4
Authentic	4
Mild to others	4
Original	4
Having a helicopter view	4
Perfectionist	4
Precise/thorough	4
Stamina	4
Responsible	4
Strong verbal skills	4
Life of the party/pacemaker	3
Powerful	3
Adaptive	3
Thinking differently	3
Capable	3
Extravert	3
Able to improvise	3
Living in the here and now	3
Acting fast	3
Resilient	3
Direct	2
Making others enthusiastic	2
Good at explaining things	2
Inspiring	2
Leader	2
Loving	2
Loyal	2
Pragmatic	2
Productive	2
Seeing things in perspective	2
Spatial awareness	2
Quick in having insight	2
Reacting fast	2
Forgiving	2
Self-esteem	2
Fine taste and hearing.	2
Other way of learning	1
Decisive	1
Experimental	1
Feel for language	1
High tolerance for frustration	1
Having a high pain threshold	1
Working in a team	1
Childish view of the world	1
Musical	1
Not a perfectionist	1
Inviting others to open up	1
Unprejudiced	1
Disarming	1
Organizational ability	1
Surrender	1
Convincing	1
Pure	1
Calm	1
Quick	1
Talking fast	1
Binding/connecting	1
Working independently	1
Meaningful	1

**Table 3:** Codes included in the qualitative analysis, their frequency and corresponding (sub)themes.

**Codes**	**Frequency**	**Subtheme**	**Theme**
Creative	159	Creative	Creativity
Energetic	63	High energy/active	Being dynamic
Enthusiastic	52	High energy/active	Being dynamic
Resourceful	48	Creative	Creativity
Empathic	44	Prosocial/empathic	Socio-affective skills
Hyperfocus	42	Attention	Higher-order cognitive skills
Associative thinking/seeing connections	41	Creative	Creativity
Driven	34	High energy/active	Being dynamic
Flexible	31	Flexible	Flexibility
Social	30	Socially outgoing	Socio-affective skills
Perseverant	29	High energy/active	Being dynamic
Imaginative	28	Creative	Creativity
Humor	25	Socially outgoing	Socio-affective skills
Spontaneous	25	Spontaneous/impulsive	Flexibility
Sensitive	21	Based on feeling/Strong emotions	Socio-affective skills
Thinking fast	21	Executive functioning	Higher-order cognitive skills
Switching fast	18	Flexible	Flexibility
Inquisitive	18	Novelty seeking/adventurousness	Being dynamic
Stress resistant	17	Other	NA
Up for anything	16	Positive attitude	Being dynamic
Image thinking	15	Executive functioning	Higher-order cognitive skills
Broad interests	15	Open	Flexibility
Seeing opportunities	15	Positive attitude	Being dynamic
Happy	13	Positive attitude	Being dynamic
Taking risks	12	Novelty seeking/adventurousness	Being dynamic
Open minded	12	Open	Flexibility
Sense of justice	12	Prosocial/empathic	Socio-affective skills
Intuitive	12	Based on feeling/Strong emotions	Socio-affective skills
Analytical	11	Executive functioning	Higher-order cognitive skills
Not boring	11	Other	NA
Sentience	11	Other	NA
Positive	11	Positive attitude	Being dynamic
Attention to detail	10	Attention	Higher-order cognitive skills
Alert	10	Attention	Higher-order cognitive skills
Enterprising/active in making plans	10	High energy/active	Being dynamic
Impulsive	10	Spontaneous/impulsive	Flexibility
Looking for challenges	8	Novelty seeking/adventurousness	Being dynamic
Supportive	8	Prosocial/empathic	Socio-affective skills
Determined	7	High energy/active	Being dynamic
Playful	7	Other	NA
Sociable/convivial	7	Socially outgoing	Socio-affective skills
Open	7	Open	Flexibility
Non-conformist	7	Other	NA
Good memory	6	Executive functioning	Higher-order cognitive skills
Intelligent/smart	6	Executive functioning	Higher-order cognitive skills
Quickly getting over fights	6	Flexible	Flexibility
Witty/sharp	6	Other	NA
Involved	6	Prosocial/empathic	Socio-affective skills
Honest	6	Other	NA
Multi-tasking	5	Executive functioning	Higher-order cognitive skills
Quickly learning/understanding	5	Executive functioning	Higher-order cognitive skills
Versatile	5	Other	NA
Sincere	5	Other	NA
Intensely enjoying /experiencing	5	Based on feeling/Strong emotions	Socio-affective skills

NA, not available. The subtheme other was not included in the themes.

#### Theme 1: Creativity

The first theme that was formed consisted of only the subtheme “creative” and had codes that specified *imaginative, creative, resourceful*, and *associative/seeing connections*. Participants in this subtheme mentioned: “Being able to think outside the box” (participant 45), “Creativity in finding solutions” (P14), “Suddenly come up with the craziest ideas that can be brilliant” (P108), “Being able to come up with surprising ideas” (P146), “Always have dreams of what could happen, a rich fantasy” (P129), and “Great problem solving ability” (P26).

#### Theme 2: Being dynamic

This theme was composed of the subthemes “high energy/active”, “positive attitude” and “novelty seeking/adventurousness”. Codes that were mentioned in this theme are *energetic, enthusiastic, up for anything* and *seeing opportunities*. Example quotes from participants for the subtheme “high energy/active” were: “Always having energy for many different things”, (P69), “Being passionate in what you do”, (P30), and “No need for coffee in the morning”, (92). Participants mentioned “Seeing opportunities”, (167)and “Not giving up”, (P36) for the subtheme “positive attitude”. The subtheme “novelty seeking/adventurousness” included quotes like “High degree of curiosity”, (P141), “Dare to take a risk even though it may not go as you hope”, (p81).

#### Theme 3: Flexibility

The theme flexibility was composed of the subthemes “spontaneous/impulsive”, “flexible” and “open” and consists of codes such as *flexible, broad interests, open minded* and *quickly getting over fights*. Participants in the subtheme “spontaneous/impulsive” mentioned “Being often very spontaneous”, (P154) and “Impulsivity”, (192). Examples of quotes mentioned in the “flexible” subtheme were “Never argue for long because I have forgotten about the cause”, (p124), “Be able to respond to changes”, (P108) and “in my work, I can switch quickly from one task to another”, (P85). The subtheme “open” included quotes like “Broad view of the world”, (P40) and “Many interests”, (P145).

#### Theme 4: Socio-affective skills

The Socio-affective theme consisted of subthemes “based on feeling/strong emotions”, “socially outgoing” and “prosocial/empathic”. Examples of codes in this theme were: *empathic, social, sensitive*, and *helping/helpful*. Example quotes in the subtheme “based on feeling/strong emotions” were “Experience life very intensely with high peaks”, (P141), “I have a huge amount of empathy and emotions”, (P158), “Sharp intuition”, (P133). Quotes mentioned in the subtheme “socially outgoing” were “I can easily make contact with others”, (P75), and “I can get along with different types of people”, (P56). For the subtheme “prosocial/empathic” participants mentioned “Standing up for others”, (P53), “Having a great sense of justice” (P110) and “I have a strong antenna for other people's feelings”, (P46).

#### Theme 5: Higher-order cognitive skills

The Higher-order cognitive skills theme consisted of the subthemes “executive functions/intelligence” and “attention”. Codes that were used were *image thinking, attention for detail, intelligent/smart* and *analytical*. Examples of quotes for the subtheme “executive functions/intelligence” were “Super-fast thinking ability”, (P14), “Good long-term memory”, (P175), “See funny stories and pictures in front of me”, (P90), and “Quick to understand”, (P188). For the subtheme “attention” sentences such as “Can focus for a long time on details”, (P11), “Can focus very well if interest is stimulated”, (P28) and “Having an eye for detail”, (P81) were mentioned.

#### Other codes

A number of codes did not fit in any of the themes and subthemes described above. Therefore, to categorize these codes we added the codes into a subtheme called “other codes”. We mention those codes here followed by one example: *playful;* “Be able to play well”, (P96), *versatile;* ”Allrounder/generalist”, (P141), *honest;* “Being honest”, (P158), *stress resistant;* “Being able to act in stressful situations”, (P144), *not boring;* “Being a bit crazy”, (P89), *sentience;* “Notice more in the environment than other people”, (P106), *sincere*; “Sincere”, (P168), *witty/sharp;* “Smart and sharp”, (P97) and *non-conformist*; “The ability to take a deviant point of view”, (P44).

#### Core symptoms of ADHD

A notable finding of our results is that the core symptom domains of ADHD, impulsivity and hyperactivity, are mentioned as positive aspects of ADHD. These symptoms, which are often a reason for individuals with ADHD to seek help, are also seen as positive aspects of ADHD by people with an ADHD diagnosis. Positive aspects such as “energetic” and “enthusiastic” were mentioned and can be linked to the hyperactivity symptoms in ADHD, and codes such as “spontaneous” and “impulsive” can be linked to the impulsivity symptoms in ADHD. The third core symptom domain of ADHD, inattention, was not mentioned as a positive aspect, but the opposite, attention (skills), such as “hyperfocus”, “attention to detail” and “alert” were perceived as positive aspects of ADHD.

### Validation of our qualitative results in a focus group

Eight individuals, five women and three men with ADHD responded to the advertisement and were invited to a focus group session. Two people canceled last minute, and therefore the focus group consisted of six participants [average age 38.66 years (*SD* = 10.54)]. The participants of the focus group reported many positive aspects of ADHD, but after checking our data we noticed that they were all already in our code list. All participants agreed that the themes and subthemes were representative of the positive aspects of ADHD.

The focus group contributed in several ways to the interpretation of the results. We identified the following topics: (1) The contribution of the results to the image of ADHD. The participants all mentioned in one way or another that the results of the qualitative analysis of the positive aspects of ADHD would contribute to a more complete picture of ADHD and that it could be helpful for many groups of people in our society to learn about these aspects (teachers, employers, peers of individuals with ADHD, family members of individuals with ADHD), example quote: “*More knowledge about positive aspects of ADHD contributes to the general image of ADHD. This information is important for teachers and employers for a better understanding of ADHD*.”, (2) Positive aspects of ADHD as a consequence of having ADHD. Participants gave examples of codes that they considered a consequence of having ADHD. For example, for the code “open-minded” a participant said “*I have always felt different from others also due to my ADHD diagnosis and therefore I am more open to others being different*”. Another example was for the code “honest”. A participant mentioned “*With all the chaos in my head due to ADHD, I am not able to handle ulterior motives or different agendas and therefore I am always honest”*. 3. The balance between positive and negative aspects of ADHD. Some of the participants agreed with the quote of one participant who mentioned “*The positive aspects will not wipe out the negative aspects of ADHD”*.

### Quantitative results

For our quantitative analyses, we calculated the frequency of the codes per code and per participant. The code *creative* was mentioned most, followed by *energetic* and *enthusiastic* (Table 2). The mean total number of codes mentioned per subject scores was 5.84 (*SD* = 4.91). Visualization of z-values showed three outliers (*z* > 3.29) that were winsorized to approach a normal distribution. The value of the outliers was replaced by the largest value. A new visual inspection showed a normal distribution of the data.

When we compared men to women on the total number of codes, we found a significant difference t_(203)_ = −2.56, *p* = 0.01, with women (*M* = 6.32, *SD* = 4.44) reporting more codes than men (*M* = 4.74, *SD* = 3.97). Looking more closely at the differences between men and women in relation to the five themes, we found women to report significantly more positive aspects in the themes *flexible* (51% females vs. 27% males, *p* = 0.001) and *socio-affective skills* (53% women vs. 34% men, *p* = 0.009) (Table 4). No correlation was found between the age of participants and the number of codes mentioned, r_(203)_ = −0.004, *p* = 0.95. No differences were found in the number of codes reported when we compared participants who reported having ever been in treatment by a clinician or coach with participants who did not receive such help, controlling for age and sex, [F_(1, 179)_ = 0.52, *p* = 0.47]. Similarly, employment status had no effect on the number of codes reported [F_(1, 164)_ = 1.86, *p* = 0.18].

**Table 4:** Sex differences across the themes of self-reported positive aspects in men and woman with an ADHD diagnosis.

**Themes**	**Number of males reporting at least 1 aspect in the theme (%)**	**Number of females reporting at least 1 aspect in the theme (%)**	***p*-value for the male female comparison**
Creativity	49 (64%)	101 (78%)	0.022
Being dynamic	42 (54%)	87 (67%)	0.073
Flexibility	21 (27%)	66 (51%)	**0.001**
Socio-affective skills	26 (34%)	68 (53%)	**0.009**
Higher-order cognitive skills	34 (44%)	56 (43%)	1.0

Reported here are the number of males and females reporting at least 1 positive aspect in each if the 5 themes. The percentages are referring to the number of males reporting a positive aspect in that particular theme relative to the total number of male participants. The *p*-values are a result of Chi-square test (2-sided) comparing male and females rations per theme. To calculate a threshold for significance that incorporates multiple testing we applied a Bonferroni correction (0.05/5 themes = 0.01). Values in bold are significant results surviving multiple comparison correction.

## Discussion

In the present study we performed qualitative and quantitative analyses on self-reported positive aspects of ADHD in a sample of 206 adults with ADHD. The qualitative analysis delivered 116 codes of positive aspects of ADHD, which were categorized into thirteen subthemes, and five themes. The five themes are *Creativity, Being dynamic, Flexibility, Socio-affective skills*, and *Higher- order cognitive skills*. We validated the results in a focus group, and participants identified themselves with the themes that were found, and the way we organized them. Our quantitative analysis showed that being creative, enthusiastic and energetic were positive aspects that were reported most. Women reported more positive aspects than men, and age, employment status and “ever received treatment by a clinician/coach” were not factors that associated with the number of positive aspects mentioned.

Compared to the qualitative studies on ADHD published before (16–18), we found both recurring and new themes in our results. The theme of *creativity* was found across all previous studies and also in our study. This, together with the result of our quantitative analysis of creativity being the most reported positive aspect, means that this is a widely recognized strength in ADHD and is not only reported in high functioning adults with ADHD but also in a more general sample of adults with ADHD. The same is true for our subtheme *high energy/active* (from our theme *being dynamic*). It is also reported in Mahdi's theme “energy and drive”, and Sedgwick's theme “energy”. Holthe's study also mentioned “high energy” and “determination”. All studies described that this is both physical energy and a source for perseverance. The subtheme *novelty seeking/adventurousness* (part of our theme *being dynamic*) was also found in Holthe's study, mentioning “risk taking” specifically. From our theme *higher order cognitive skills*, and *subtheme attention*, hyperfocus is a concept that was also found in Mahdi's and Sedgewick's studies. The theme *socio-affective skills* with the subtheme *prosocial/empathic*, comes back in the related themes of “agreeableness” and “willingness to assist others” in Mahdi's results and as “humanity” in Sedgewick's results. (Sub)Themes of positive aspects that, to our knowledge, were found for the first time in our study, are the theme *flexibility*, and the subthemes *positive attitude, strong emotions*, and *executive functions*. This could be due to the relatively large sample that we included which likely captured more positive aspects covering a wider spectrum due to the larger variation of individuals in our sample. In contrast, Sedgwick's study used a sample of 6 high functioning males and Holthe's study included 5 successful women. Themes that were found in Sedgewick's article, but did not come up in our results, are “appreciation of beauty and excellence”, and “sublimation” (cognitive reappraisal). This could possibly be due to the differences in participants between the studies. Sedgewick's study included high functioning and highly educated adults who might have more access to art and cultural aspects of life than our community sample. A difference in research methods could also explain this discrepancy as in Sedgwick's study they performed extensive interviews whereas we mainly had one-word quotes and sometimes a short sentence. This might have led to themes with more depth such as the theme of sublimation. Finally, cultural differences could explain differences between samples, as some strengths in one country or culture might not be considered strengths in another country/culture (22).

### Quantitative analysis

In general, almost all our participants experienced one or more positive aspects of ADHD. Unlike previous studies, we did not only focus on highly successful individuals with ADHD, therefore we can conclude that experiencing positive aspects is common in ADHD, rather than reserved to a minority that is highly successful.

Our quantitative analysis showed that creativity was the most frequently mentioned positive aspect of ADHD. This may indicate that individuals with ADHD consider themselves to be more creative than others. Previous research using quantitative studies has confirmed a positive association between ADHD and creativity, although not all aspects of creativity are associated with ADHD. Divergent thinking shows associations with ADHD but not convergent thinking (19). When we zoom in on our theme of creativity we find *imaginative, creative, associative thinking/seeing connections*, and *resourceful* and indeed notice that these are aspects that are linked to divergent thinking.

We identified differences between men and women in reporting positive aspects of ADHD. Women reported more positive aspects than men. A reason for this could be that women in general give longer answers to open questions (23, 24). Another difference between men and women, is that women tend to be more aware of symptoms (25, 26). There is also evidence for women in general to report more, and score higher on, character strengths than men (27). The difference between men and women in our study was specifically seen for the themes *socio-affective skills* and *flexibility*. This is an interesting finding as socio-affective skills, like social support and social relationships, seems to be an area where adolescent women with ADHD often struggle. For example, the lack of confidence in relationships and having fewer friends (28, 29). Our themes are very broad and future quantitative research should further specify sex effects in relation to positive aspects of ADHD.

Being employed vs. unemployed, and being in treatment by a clinician (past or present) vs. never having been in treatment by a clinician/coach, did not affect the rate of positive aspects mentioned by individuals with ADHD. We assumed that participants who were not employed or who were receiving disability pensions would possibly be more impaired by their ADHD and therefore might not experience many positive aspects. We also considered that receiving treatment in past or present would lead to increased quality of life and that therefore more positive aspects would arise. Unfortunately, we do not have any additional data on impairment or quality of life in our sample to further unravel these findings. To better understand what it means to report many positive aspects, future research should be dedicated at studying the relationship between the frequency of reported strengths and wellbeing, impairment and quality of life. In addition, we should acknowledge that our treatment variable gave us limited information, for example, we could not specify pharmacological or non-pharmacological treatment. Previous work has provided evidence for pharmacological treatment not affecting the most reported positive aspect, creativity (30). However, this can vary for each individual positive aspect. Something else to consider is the thought that people with ADHD who chose not to receive any kind of treatment, might have made this choice in light of a neurodivergent perspective of ADHD, embracing their ADHD, and experiencing benefits. This would lead to increased reports of positive aspects in the group of the untreated individuals and might counter balance the positive aspects in the treated group. This line of thought is very speculative, however, it highlights that we are currently unaware of how sentiments and perspectives of ADHD affect the (frequency of) reported positive aspects of ADHD in people with ADHD. A more general thought about our sample is that our participants, members of an organization for ADHD, might be especially engaged with their ADHD and therefore in general may be more aware of aspects of ADHD, including the positive aspects.

In all, future studies researching positive aspects in ADHD should collect more demographic and clinical information, information about wellbeing and indices of quality of life of the individuals in the study, and possibly also information about the believes and sentiments of ADHD to further our understanding of positive aspects in ADHD.

### Are positive aspects inherent to ADHD, or coping mechanisms, or something else?

The current study has delivered a list of self-reported positive aspects of ADHD. These were grouped based on the relatedness of the aspects in terms of the meaning of the aspects. There is also the possibility to make a different distinction, based on what the participants of the focus group suggested. For example, there might be positive aspects that are a consequence of living with ADHD such as being open-minded, honest or perseverant. Due to the more advanced age of our sample, we very likely have also captured these positive aspects related to ADHD, that are a result of coping with ADHD. Thus far, little is known about the longitudinal course of positive aspects of ADHD. Future research should aim to explore strengths in individuals with ADHD from an early age, this way, coping mechanisms as a result of having ADHD can be excluded or eliminated.

Another group of aspects could be defined as being the counterpart of the deficit aspects of ADHD, originating from the same underlying neurobiological mechanism. This was also proposed in the Dynamic Developmental Model, where adaptive and maladaptive outcomes are evaluated in relation to symptoms of ADHD (31). Here one could think of divergent thinking, the ability to come up with novel ideas. For divergent thinking ongoing thoughts are essential (adaptive). The mechanisms associated with these ongoing thoughts could overlap with the mechanisms that are associated with response inhibition problems (not being able to inhibit ongoing thoughts/processes), often present in individuals with ADHD and which are generally considered maladaptive (32). Studies investigating positive aspects as well as cognitive deficits in one sample may reveal how positive and negative aspects of ADHD coexist and are associated with each other.

There is also a group of aspects that have, depending on the situation, either a negative consequence or a positive consequence. For example, impulsivity and related characteristics such as spontaneity and novelty seeking, are considered positive aspects of ADHD but are also part of the core ADHD symptoms as defined by international classification systems such as the DSM-5. Dependent on the situation the same characteristic may be beneficial and positive, or detrimental. For instance: acting impulsively in a situation of crisis can be beneficial. Future research using for example digital applications could monitor aspects such as impulsivity throughout the day and identify in which situation impulsivity is beneficial or detrimental. More insights into the situational aspects can lead to better management of these aspects of ADHD and help find a balance between beneficial and detrimental effects.

From the above it becomes clear that we do not know much about where the positive aspects originate from. Do they develop together with core ADHD symptoms, or are they causal to having an ADHD diagnosis? There might even be more conceptual groups of positive aspects of ADHD than just the three mentioned above. From the perspective of psychoeducation, it is of high relevance to perform research into the development of the positive aspects of ADHD, as one could imagine that there is a distinction between addressing positive aspects from the “causal” category (e.g., “because of your ADHD you are open-minded”), from the “positive counterpart” category (e.g., “you are very good at thinking outside the box, but this goes together with your inability to inhibit responses”) or from the “context/situational” category of aspects (e.g., “in certain circumstances you are bothered by your impulsivity but in other circumstances you can benefit from it”).

### Implications

Our findings align with current clinical guidelines recommending a multidisciplinary approach for treatment of ADHD, with a range of non-pharmacological treatment options alongside medication if necessary (33). Our data demonstrate that core symptoms of ADHD such as impulsivity may represent a problem but also a strength. Therefore, it is crucial that clinicians and people with ADHD collaborate to find a personal balance that works for that particular person with ADHD in their particular situation and stage of life, to maximize quality of life.

Finding positive aspects or strengths of individuals with ADHD might also provide other new perspectives for healthcare. First, assessing strengths could become part of the diagnostic process. Ideally, one or more questionnaires could be developed assessing ADHD specific strengths, providing both self and other reports. Second, it paves the way for strength-based interventions and narrative interventions based on positive psychology, complementary to interventions based on symptom reduction. Strength-based interventions have the potential to contribute to beneficial outcomes in various areas of life (34). Previous meta-analyses have shown that these interventions help decrease depression, anxiety and stress and increase life satisfaction and quality of life (34, 35). Strength-based and neurodiverse perspectives have been used in Autism Spectrum Disorder before with promising results (36).

For individuals with ADHD, learning more about the positive aspects associated with ADHD can have a major impact on coping with their condition, for example, *via* improving their self-esteem, life satisfaction, wellbeing and health related quality of life. Previous research has shown that strength-use predicts wellbeing and quality of life in the general population (37) and that specific for ADHD, strengths such as “being sensitive toward other people's emotions” and “being tolerant and open” show a positive correlation with self-esteem (12). The results of our study can contribute to existing and to developing new strength-based interventions in ADHD.

The identification of positive aspects of ADHD may also contribute to a changed perspective of ADHD in society, from an exclusive “deficit” way of approaching individuals with neurodevelopmental conditions, toward embracing inclusivity, accommodating differences, and taking advances of diversity. For example, research showed that having ADHD is meaningful for entrepreneurship (38–40). Identifying a broad spectrum of positive aspects associated with ADHD and transferring this knowledge to employers and teachers, will increase the chances for people with ADHD to reach their full potential.

### Strengths and limitations of the current study

Our study has several strengths and limitations. A strength of the current study is the sample size: to the best of our knowledge, this is the largest sample size to date reporting on positive aspects of ADHD. This allowed for identification of a large number of positive characteristics and performing quantitative analysis on those aspects. For example, it allowed us to give an indication of which strengths are more prevalent than others. Due to the larger sample size, our sample was more diverse than previous qualitative studies, and therefore our results provide a more diverse coverage of positive characteristics. A limitation is recruitment bias: people who suffer severely from ADHD symptoms may not be members of the Dutch organization for people with ADHD. For example, the rate of employment in our sample (60.3%) was much higher than the employment rate in a Norwegian sample of individuals with ADHD (24.3%). More than one third of their sample received disability benefits compared to only 8% in our sample (41). A Swedish registry study showed that 12 % of individuals with an ADHD diagnosis received disability pensions (42). In addition, 60% of our study sample was female while research in national registry samples show a higher prevalence of adult ADHD in males (41). Our quantitative results showed a significant difference between men and women in the number of positive aspects, with women mentioning more aspects than men. This may implicate that the number of positive aspects mentioned in our study may be an overrepresentation due to gender bias; future studies should aim for equal gender distribution. Finally, the response rate of our study was 26%, which means that for the larger part of the group of members of the organization, the perception of positive aspects is unknown.

Another strength of our study was the participatory evaluation of survey data by members of the focus group. This provided us with meaningful new interpretations of our findings, such as the different categories that might exist within the list op positive aspects that were reported. Participatory research in general has been shown to be advantageous for both researchers and participants, because it leads to more relevant results for both groups (43).

A limitation of our study is the lack of clinical and demographic information of our participants. We did not have information on level of functioning, type and severity of ADHD symptoms, and presence of co-existing conditions (other than dyslexia/dyscalculia). It could be hypothesized that – for example - individuals with ADHD and co-existing depression, may report fewer positive aspects. A study design that incorporates demographic, social and clinical data may explore correlations between clinical characteristics, level of functioning and number and type of positive connotations with ADHD.

In the current study, we researched *self-reported*- and therefore subjective - positive aspects. Our study does not provide evidence for individuals with ADHD being indeed more *driven, empathic*, or have more *humor* (random examples) than individuals without ADHD. Empathy, for example, was reported as a positive aspect of ADHD in our study but also in Mahdi's study, and in quantitative studies investigating self-reported and observant reported empathy was lower in children and adults with ADHD (44–48). Therefore, future research should be aimed at validating the list of positive aspects by means of quantitatively and objectively assessing positive aspects in individuals with and without ADHD. We do expect it to be challenging to find measurements that exactly capture the positive aspects that come from self-report qualitative studies.

Finally, asking people about their qualities, is asking them to share their self-image. This study did not investigate the influence of the prevailing opinion about ADHD which likely affects their self-image and the positive aspects that they reported. Further studying opinions about ADHD and self-image in relation to positive aspects of ADHD will provide an opportunity to disentangle those aspects and will therefore generate a clearer picture of positive aspects of ADHD.

## Conclusion

We have provided confirmation of self-reported positive aspects of ADHD found in other studies, and we identified new self-reported positive aspects of ADHD. As almost all participants reported positive aspects of ADHD, we concluded that experiencing positive aspects is common in our cohort, which – together with earlier research – makes it probable that experiencing positive aspects are common in people ADHD. The positive aspects that have been reported cover many different domains and can serve as direct input for studies with ecologically valid approaches (go beyond self-report) to identify strengths in ADHD. We need for example quantitative studies with measurements of these strengths and studies using other-report before we can start thinking about incorporating specific strengths into the image of ADHD. The identification of strengths in ADHD can provide implications for the management of ADHD. Awareness about ADHD strengths can help individuals with ADHD and their environment to better cope with, accept or embrace their diagnosis and for example make educational or occupational choices that fit them well. To incorporate these positive aspects in the understanding of ADHD, future research should also focus on investigating the link between these aspects and clinical characteristics and how this new knowledge can be implemented in psychoeducation and find its way to education and occupational settings.

## Data availability statement

The datasets presented in this article are not readily available because we do not have consent from our participants to share the data. Data are in Dutch. Please contact the corresponding author for information about using derived data. Requests to access the datasets should be directed to martine.hoogman@radboudumc.nl.

## Ethics statement

Ethical review and approval was not required for the study on human participants in accordance with the local legislation and institutional requirements. The patients/participants provided their written informed consent to participate in this study.

## Author contributions

HV, RP, JM, CG, and MH initiated, designed and set-up the study. LS, LH, and MH performed the analysis. JM, CG, and MH reviewed and debated the qualitative results. LS, LH, and MH organized the focus group session with help from HV and RP. LS, LH, JZ, and MH wrote the manuscript with active contributions from all coauthors. All authors contributed to the article and approved the submitted version.

## Funding

MH is supported by a personal Veni grant from the Netherlands Organization for Scientific Research (NWO, Grant No. 91619115).

## Conflict of interest

The authors declare that the research was conducted in the absence of any commercial or financial relationships that could be construed as a potential conflict of interest.

## Publisher's note

All claims expressed in this article are solely those of the authors and do not necessarily represent those of their affiliated organizations, or those of the publisher, the editors and the reviewers. Any product that may be evaluated in this article, or claim that may be made by its manufacturer, is not guaranteed or endorsed by the publisher.
